# Exploring care staff perspectives of malnutrition in residential aged care

**DOI:** 10.1111/ajag.70054

**Published:** 2025-06-11

**Authors:** Karly Bartrim, Rhiannon Hill, Marie‐Claire O'Shea

**Affiliations:** ^1^ School of Human Movement and Nutrition Sciences The University of Queensland St Lucia Queensland Australia; ^2^ School of Health Sciences and Social Work Griffith University, Gold Coast Southport Queensland Australia

**Keywords:** caregivers, homes for the aged, malnutrition, residential aged care

## Abstract

**Objective:**

This study aimed to explore the understanding of care staff in Residential Aged Care (RAC) regarding malnutrition and their perspectives on the multideterminant factors associated with providing nutrition care to residents.

**Methods:**

A qualitative study was conducted, utilising the socioecological model as a framework. Focus groups were conducted with current RAC care staff and were recruited via convenience sampling. Three focus groups were conducted using semi‐structured questions.

**Results:**

Fifteen RAC care staff (*n* = 13 female participants) with a range of career experience participated in focus groups (five participants per focus group). Two main themes and five subthemes were identified. Theme 1: lack of understanding of malnutrition, with subthemes of: 1.1: there are no residents with malnutrition in my facility, 1.2: gaining weight is more of a concern than weight loss (associated with malnutrition), and Theme 2: feeling restrained in doing more, with subthemes of: 2.1: limited time and lack of staff impact the care I can provide, 2.2: residents with malnutrition have greater care needs, and 2.3: lack of social support contributes to malnutrition.

**Conclusions:**

The study explored malnutrition issues in RACs from care staff perspectives. Care staff expressed genuine care for residents, yet felt they lacked capacity to provide adequate support, particularly for those with malnutrition. Staff shortages, inadequate training, and time constraints affected their ability to attend to resident needs. The study identified systematic issues, particularly at the policy level, in malnutrition management, providing a clear focus for improvement and future research.

**Practice impact:**

Care staff require more training to enhance their understanding of malnutrition and therefore adequately support residents living with malnutrition in residential aged care homes. Care staff emphasise the desire to provide genuine care to all residents and recognise that residents with malnutrition have higher care needs.

**Policy impact:**

Increasing social support for residents is considered essential by care staff, to reduce malnutrition risk. To build care staff workforce capacity, changes at the policy and residential aged care home level are needed, with a focus on ensuring care staff are provided adequate nutrition training and sufficient time to care for residents with malnutrition.

## 1 INTRODUCTION

The global population is ageing,^1^ with the number of individuals over 85 years of age in Australia predicted to double in the next 30 years to an estimated 1.7 million people.^2^ With an ageing population, the demand for Residential Aged Care (RAC) settings is expected to rise.^3^ In 2018, the Royal Commission into Aged Care Quality and Safety announced concerns for quality and safety of care provided in RACs. The final report, published in 2021, indicated 68 per cent of RAC residents in Australia were at risk of or had malnutrition, calling for ‘immediate action’ to address this crisis.^3^

While malnutrition is not a normal part of ageing, older adults are at greater risk due to physiological and environmental changes.^4^ Malnutrition refers to the deficiencies or imbalances in a person's intake of energy and/or other nutrients, particularly associated with undernutrition.^4^ The underlying causes of malnutrition can be diverse, multifactorial and vary from person to person.^4^ The most common risk factors are often referred to as the ‘nine Ds’, which encompass poor dentition, dysphagia, dysgeusia, diarrhea, depression, disease, dementia, dysfunction and drugs, many of which are present or experienced by residents within RAC.^4^ Malnutrition in older adults has significant consequences, including high hospital readmission rates, functional and cognitive decline, reduced muscle function and atrophy, decreased immune function, increased risk of pressure injuries and higher mortality rates.^5^, ^6^ Given the adverse effects of malnutrition on health and well‐being,^7^ all staff in RAC have a role in malnutrition prevention and management.

Care staff, also known as personal care workers (PCW), personal care assistants (PCA) and assistants in nursing (AIN), are typically considered the frontline in RAC.^8^ Care staff currently make up approximately 70% of the RAC workforce in Australia.^8^ As such, care staff have a diverse role, with assisting residents in activities of daily living, such as food provision, mealtime support, feeding assistance, and personal hygiene tasks.^8^, ^9^ Despite the central role of care workers in residents' care, in Australia, there is no minimum standard qualification for these roles.^10^ Additionally, those with vocational or certificate qualifications are unlikely to have received any training regarding food provision, nutrition care, mealtimes or about the condition of malnutrition within their training.^11^ Care staff are core members of the RAC team and play a crucial role in providing nutrition care or support to older adults.^11^

Despite the central role of care staff in providing support to residents to improve their nutrition and reduce the risk of malnutrition,^11^ no research to date has explored the perspectives of care staff on nutrition care or malnutrition. Research to date has specifically focused on registered nurses' knowledge, attitudes and perspectives towards malnutrition in RAC,^12^, ^13^ or a combination of staff perspectives, which includes care workers.^14^, ^15^ With care staff making up a large proportion of the RAC workforce and the high prevalence of malnutrition in aged care, there is an opportunity to explore care staff perspectives on malnutrition. It is imperative to explore this knowledge gap to identify opportunities for future research and develop solutions to improve the high prevalence of malnutrition in RAC.^12^, ^13^, ^16^ Therefore, this study aimed to explore the understanding of RAC staff regarding malnutrition and their perspectives on the multideterminant factors associated with providing nutrition care to residents.

## 2 METHODS

### 2.1 Study design and theoretical framework

This descriptive qualitative study used the socioecological model (SEM) as a framework.^17^ The SEM is a comprehensive framework that recognises the various determinants of health, encompassing individual, interpersonal, organisational, community and political factors.^17^ Previous studies in the aged care sector, such as a study conducted by Mah et al. (2021), have employed the SEM, enabling a holistic qualitative research.^18^ More specifically, a qualitative study exploring frontline staff (i.e. diet and nursing aids) perspectives of their roles in food and nutrition care acknowledged structural and organisational constraints or changes impacting their ability to deliver resident food and nutrition care, especially to those with malnutrition.^14^ As such, this highlights that there is a need to explore the broad range of determinants related to malnutrition through the perspectives of care staff.^14^ The SEM framework was chosen to guide this study due to its application in prior aged care studies,^18^ as well as the multifactorial determinants of malnutrition^14^ and factors that can impact care staff roles.^19^

Ethics approval was obtained from the Griffith University Human Research Ethics Committee (GU HREC: 2022/372). This manuscript adheres to the Consolidated Criteria for Reporting Qualitative Research (COREQ).^20^ The research team included a student dietitian and two dietitian–researchers (with experience working and researching in RAC). A team of experienced nurse‐researchers also provided input into study design and interview questions.

### 2.2 Study setting, participants and recruitment

Focus groups were held at three different RAC settings located in New South Wales and Queensland, Australia. A convenience sample of RAC homes were chosen based on existing relationships from previous malnutrition research.^21^ In the previous year, each site's manager collaborated with student dietitians to screen all residents for malnutrition, revealing that 3% had severe malnutrition and 31% were at risk of/had mild‐to‐moderate malnutrition.^21^

The study participants were care staff employees of the participating RAC sites. No specific exclusion criteria were applied. Convenience and snowball sampling were used to recruit participants, through a member of the research team contacting RAC managers. The managers then sent an email to care staff in their RAC site, which included study details, the participant information sheet and researcher contact information if interested in participating. Each participant received a $50 Coles/Myer gift card as an honorarium. Due to funding limitations, a maximum of 15 participants across all focus groups could participate.

### 2.3 Data collection

Three separate semi‐structured focus groups were conducted with care staff to explore their perspectives of malnutrition within RAC. Focus groups A and B were conducted by one research team member via Microsoft Teams™, supported by two other researchers who attended in person to facilitate the focus groups. Focus group C was conducted in person with two research team members. Before each focus group, all participants provided both written and verbal consent to being involved in the focus group and for the session to be recorded. Sessions were recorded and automatically transcribed via Microsoft Teams™. One member of the research team reviewed each transcript and cross‐checked the details for accuracy. While providing the opportunity for participants to review the transcripts is important to enhance the quality and rigour of the results,^22^ it was declined due to their work commitments and the limited time allocate to be involved in the research.

Using the SEM framework,^17^ the research team developed a focus group guide (Table 1), that included an inquiry purpose, nine questions and probing questions.^23^ The guide was used in all focus groups to maintain uniformity and consistency. The guide was then pilot‐tested by three individuals with vocational qualifications. Demographic information, including age, employment type and years of experience, was collected at the start of the focus group by a member of the research team.

**TABLE 1:** Focus group interview guide used to explore RAC care staff understanding of malnutrition, and their perspectives on the multideterminant factors associated with providing nutrition care to residents.

Socioecological model framework level—Enquiry purpose	Interview questions	Potential probing questions
Individual level—To understand care staff's awareness of residents with malnutrition and how they consider malnutrition may present	Can you describe a resident that you care for who has malnutrition? How do you know they have malnutrition?	What are some signs of malnutrition? What is your understanding of malnutrition (or the impact of low body weight) on a resident?
Individual level—To understand the care staff's role in providing nutrition care to residents with malnutrition and the processes involved in malnutrition management	3 Can you describe how you care for residents with malnutrition in relation to their nutrition needs? 4 What is your role/ responsibility in relation to malnutrition identification and management?	What would you do if you noticed someone who appears to have malnutrition? Who communicates if a resident has malnutrition to you /who do you contact? What is your role in providing nutrition care to residents with malnutrition?
Interpersonal level—To understand care staff's perceptions on the impact of malnutrition on their role and how malnutrition may impact the resident and/or their families	5 What impact does malnutrition have on your resident? 6 What impact does malnutrition have on you as a carer for the resident? 7 Can you describe families involved in providing nutrition support or assistance during mealtimes?	Do residents with malnutrition change the way you provide care? Do residents with malnutrition change your workload? Does the involvement of family support your role as a carer or change residents' care?
Organisation/community/policy level—To understand care staff's perceptions of the influences of the RAC they work in, the RAC provider, the aged care sector more broadly and Government policy in providing nutrition care and malnutrition prevalence	8 What helps or hinders your ability to deliver nutrition care as part of your routine everyday care for residents? 9 The Royal Commission in Aged Care Quality and Safety revealed that malnutrition is highly prevalent and a huge concern in aged care. How do you think malnutrition in RAC be reduced?	What are some challenges of delivering nutrition care as part of your role? How can you be better supported in your role to assist in reducing malnutrition prevalence?
	Conclusion: Provide the definition of malnutrition at the end of the interview, ask about their awareness of the previous research study, share the findings of malnutrition prevalence in their RAC, and provide the opportunity for comments, questions or reflections	

### 2.4 Data analysis, rigour and trustworthiness

The data were thematically analysed using the Braun and Clarke six‐step methodology.^23^ The Braun and Clarke methodology was chosen to systematically organiswe, describe and interpret the data. Given that the research question has not yet been explored in existing literature, the use of inductive methodology is well‐supported.^24^ One researcher (RH) read and re‐read each transcript, then line‐by‐line coding was conducted, and codes were added into a table to generate prospective thematic concepts. To strengthen the reflexivity, credibility and defensibility of the analytical process,^22^ the other members of the research team cross‐checked each step and sense‐checked through reflecting and debriefing regularly with the researcher analysing the data. The research team then met to refine the themes, which involved reviewing, defining and naming themes. Results are reported by themes, using quotes within an analytical narrative to answer the research question.^24^ Each participant was assigned a unique code consisting of the letter ‘P’ followed by a number and the focus group location (A, B or C). Descriptive statistics of count and per cent were used to report the study participants' demographic data.

## 3 RESULTS

Three focus groups were conducted in January 2023 across three RAC sites. On average, each focus group lasted approximately 34 min. Fifteen participants attended the focus groups, with five participants in each group. Participants were mostly women (86%, *n* = 13), and their ages ranged from 16 to 59 years old. All participants were employed as care staff and had varying years of experience (0–20 years). All care staff involved in the focus groups had vocational‐level training. Table 2 summarises the demographic characteristics of the focus group participants.

**TABLE 2:** Demographic characteristics of focus group participants (*n* = 15).

Characteristics	Focus group A, *n* (%)	Focus group B, *n* (%)	Focus group C, *n* (%)	Total participants, *n* (%)
Gender				
Female	5 (100)	5 (100)	3 (60)	13 (86)
Male	0 (0)	0 (0)	2 (40)	2 (14)
Age				
16–29	0 (0)	2 (40)	0 (0)	2 (13)
30–39	1 (20)	0 (0)	3 (60)	4 (26)
40–49	0 (0)	1 (20)	1 (20)	2 (13)
50–59	1 (20)	2 (40)	1 (20)	4 (26)
60–69	3 (60)	0 (0)	0 (0)	3 (20)
Years of experience				
0–4	1 (20)	1 (20)	1 (20)	3 (20)
5–9	2 (40)	2 (40)	1 (20)	5 (33)
10–14	1 (20)	1 (20)	3 (60)	5 (33)
15–20	1 (20)	1 (20)	0 (0)	2 (13)
Employment type				
Permanent full‐time	0 (0)	2 (40)	1 (20)	3 (20)
Permanent part‐time	0 (0)	3 (60)	3 (60)	6 (40)
Permanent casual	5 (100)	0 (0)	1 (20)	6 (40)

Two main themes and five subthemes were identified (Table 3). Theme 1: lack of understanding of malnutrition, with subthemes of: 1.1: there are no residents with malnutrition in my facility, 1.2: gaining weight is more of a concern than weight loss (associated with malnutrition); and Theme 2: feeling restrained in doing more, with subthemes of: 2.1: limited time and lack of staff impact the care I can provide, 2.2: residents with malnutrition have greater care needs and 2.3: lack of social support contributes to malnutrition.

**TABLE 3:** Overview of themes, subthemes and key points identified, exploring RAC care staff's understanding of malnutrition and their perspectives on the multideterminant factors associated with providing nutrition care to residents.

Themes	Sub‐themes	Key points identified
1. Lack of understanding of malnutrition	1.1. There are no residents with malnutrition in my facility	Care staff did not perceive malnutrition as an issue among the residents in their care There was a lack of understanding about the signs and symptoms malnutrition and how it may be assessed, as care staff were unable to identify residents with diagnosed malnutrition in their RAC site Participants described residents with malnutrition as those who ‘looked thin’
	1.2. Gaining weight is more of a concern than weight loss (associated with malnutrition)	Participants expressed a greater worry about residents gaining weight when coming into their facility Care staff described gaining weight as a greater concern that affects more residents than weight loss associated with malnutrition The concerns regarding weight were linked to the high frequency of meals provided within RAC and that often residents enter RAC from home, where they may have not been cooking and eating as much
2. Desire to do more, but recognising the challenges of providing optimal nutrition care	2.1. Limited time and lack of staff impact the nutrition care I can provide	Emphasised the impact of inadequate staffing and the limited time they have to provide care to residents Care staff acknowledged that a resident with malnutrition has greater care needs, which further impacts their workload and their ability to provide timely adequate care Participants expressed concerns about limited staffing capacity within their RAC site, which they felt directly impacted their ability to provide adequate care for residents There was a shared view among the participants regarding the need for increased staffing and greater capacity to fulfil their caregiving roles Participants linked understaffing to reduced care time, expressing a sense of just ‘doing the job’ without being able to provide genuine care Some participants expressed concern about not being able to engage with residents or make a meaningful difference
	2.2. Residents with malnutrition have greater care needs	Care staff described and reflected on prior experiences of caring for residents with malnutrition (mainly those with malnutrition and are nearing end‐of‐life) Care staff conveyed the genuine care and empathy they provide to residents in their care The overall expressed by many participants was a sense of helplessness Despite the care staff acknowledging residents' choice, there was an underlying obligation or need to continue to care and support the residents Care staff described ownership of caring for residents' nutrition and that they would take it on themselves to report it to more senior staff they noticed anything or were concerned
	2.3. Lack of social support contributes to malnutrition	The impact of a lack of social support for residents was mentioned as a key contributor to malnutrition Participants agreed that poor social support, can result in feelings of loneliness or poor mental health, can result in a loss of motivation to eat, leading to malnutrition Participants recognised residents' need for social connections and expressed concerns about the lack of support provided to residents Participants highlighted the role of families in providing support which directly impacts nutritional intake

### 3.1 Theme 1: Lack of understanding of malnutrition

#### 3.1.1 Sub‐theme 1.1: There are no residents with malnutrition in my facility

Throughout the focus groups, care staff revealed a limited understanding of malnutrition. Participants did not perceive malnutrition as an issue among the residents in their care. It was clear among the majority of participants that there was a lack of understanding about the signs and symptoms of malnutrition and how it may be assessed, as care staff were unable to identify residents with diagnosed malnutrition in their RAC.^25^ When asked to describe a resident with malnutrition they cared for, participants in two of the three focus groups responded with statements such as, ‘we haven't had any’ [P3A]. There was general agreement among participants that residents with malnutrition ‘looked thin’. Many care staff associated malnutrition with residents who are end‐of‐life, with one participant stating, ‘I think the only time we've really seen it (malnutrition) here is really end of life’ [P2A]. Another participant reflected back to ‘when I was a student’ [P5A] in order to recall a resident with malnutrition, indicating their belief that malnutrition is a rarity. Only one participant was able to describe a resident they had cared for who appeared very thin, stating, ‘She (resident) was literally a skeleton inside skin’ [P5C], but did not specifically describe the resident as having malnutrition. In the same focus group, a different viewpoint was presented, with the participant expressing, ‘Just saying old skinny people are malnourished, I don't think so. I think that's a misconception’ [P4C]. Furthermore, one participant described non‐physical aspects of malnutrition, mentioning someone who ‘Sleeps a lot and has no energy’ [P4B], but again, did not recall a resident with malnutrition in their care.

#### 3.1.2 Subtheme 1.2: Gaining weight is more of a concern than weight loss (associated with malnutrition)

Participants expressed a greater worry about residents gaining weight when coming into their facility and described it as a greater concern that affects more residents than weight loss associated with malnutrition. One participant emphasised the issue by stating, ‘Most people that come [into RAC] actually put on weight. That's where we get problems’ [P1A].

Others shared similar opinions, with one participant stating, ‘Yep, to me it's my biggest problem here’ [P3A]. The concerns about weight were linked to the high frequency of meals provided within RACs—‘Breakfast, morning tea, lunch, afternoon tea, supper… Sometimes they just keep eating’ [P2B]—and that often residents enter RAC from home, where they may have not been cooking and eating as much.

### 3.2 Theme 2: Feeling restrained in doing more

#### 3.2.1 Subtheme 2.1: Limited time and lack of staff impact the nutrition care I can provide

Time poor staff and chronic staff shortages were strong themes throughout all focus groups. Beyond providing care to residents with malnutrition, all participants in the focus groups emphasised the impact of inadequate staffing and the limited time they have to provide care to residents. More specifically, care staff acknowledged that a resident with malnutrition has greater care needs, which further impacts their workload and their ability to provide timely, adequate care. Care staff stated:

‘Our job can become more demanding. Sometimes we might need two staff to look after them (residents), two staff to stand them up.’ [P4B]

‘I know this sounds horrible, but it (malnutrition) impacts the staffing as well. So, if we had a person with malnutrition that's not feeling too well… staff can't act timely. When you've only got two staff members having more would make the difference.’ [P1C]

‘Creates a lot more work if they're in physical decline because of their nutrition.’ [P5A]

Participants expressed concerns about limited staffing capacity within their RAC, which they felt directly impacted their ability to provide adequate care for residents. There was a shared view among the participants regarding the need for increased staffing and greater capacity to fulfil their caregiving roles, as one participant stated, ‘We need to have people.’ That's the issue. Well, sounds really simple, we need the numbers’ [P5A]. Another participant echoed this opinion, stating, ‘We don't have the people’ [P5C].

Participants linked understaffing to reduced care time, expressing a sense of just ‘doing the job’ without being able to provide genuine care. One participant reflected on this, stating, ‘I know that sounds really bad. But sometimes I feel like I'm coming in and I'm like a robot’ [P4B]. Another participant expressed concern about not being able to engage with residents or make a meaningful difference, stating, ‘I'm not having a chat with them, trying to engage with them’ [P5B].

#### 3.2.2 Subtheme 2.2: Residents with malnutrition have greater care needs

Even though participants in the focus groups could not identify current residents with malnutrition when prompted, care staff described and reflected on prior experiences of caring for residents with malnutrition (mainly those with malnutrition and are nearing end‐of‐life). Care staff conveyed the genuine care and empathy they provide to residents in their care. One participant displayed emotion while discussing their relationship with the residents, stating:

‘If you've known them a long time and you can see the decline… it can be like a really big impact on you. Especially if you become close with them and you can see it happening and you do all you can… but when you can't help them, and you can see it happening, that's hard.’[P4B]

The overall sentiment expressed by many participants was a sense of helplessness, ‘We just wish we can help them with what they are going through’ [P1B].

Despite the care staff acknowledging residents' choice (‘It's their [resident's] choice to eat and to drink. And we can't make them eat and drink’ [P4A]), there was an underlying obligation or need to continue to care and support the residents; ‘If somebody was really declining in their weight. Yeah, it would be left up to us care staff to fix’ [P2A]. Care staff used words such as ‘our mission’ [P1A] to ‘encourage’ [P2A] to describe their role in nutritional care and stated they would take it on themselves to have it ‘reported higher up the chain and then they contact the families’ [P5A] if they noticed anything or were concerned.

#### 3.2.3 Subtheme 2.3: Lack of social support contributes to malnutrition

The impact of a lack of social support for residents was discussed among many participants as a key contributor to malnutrition. Participants agreed that poor social support can lead to feelings of loneliness or poor mental health, resulting in a loss of motivation to eat, leading to malnutrition. One participant said:

‘I think mental health does come into play with how they feel about being away from their family, not being in their own home, stuff like that. They just stopped being some of them just stop eating here, because they're like, what's the point?’ [P4C]

Participants recognised residents' need for social connections and expressed concerns about the lack of support provided to residents. One participant stated:

‘I think most of them are lonely. Some of them don't have any family. Some of them don't have that many people to come and see them. I just feel that they are giving up.’[P5B]

Another participant highlighted the role of families in providing support, which directly impacts nutritional intake, stating:

‘I think that all depends on the individual and the different types of families. We have some families that are just absolutely wonderful. They've bent over backwards; they bring in all sorts of goodies and beautiful fruits and what have you. Some residents here have no family at all. And if they do, they don't visit.’ [P1A]

## 4 DISCUSSION

This study provided the first insights into the understanding of RAC care staff regarding malnutrition and their perspectives on the multideterminant factors associated with providing nutrition care to residents. The findings of this study suggest that care staff may be unable to identify residents with malnutrition or may not consider the impact of weight loss associated with malnutrition as the biggest nutritional issue in RAC. While it appears that care staff lack understanding of the prevalence of malnutrition and its associated risks to residents in RAC, care staff identified the multifactorial causes of malnutrition and their ability to provide nutrition care to residents. Care staff acknowledged the increased care needs of residents who have malnutrition, the link between social support and malnutrition, as well as the impact of limited staff capacity on providing care to residents with malnutrition. The findings of this study highlight the need to build the capacity of the care staff workforce to ensure care staff can provide genuine care to residents living with malnutrition in RAC.

The study highlighted a gap in care staff understanding with respect to what malnutrition is, as care staff were unable to identify residents in their care who had malnutrition. In addition, care staff revealed poor understanding of nutrition‐related priorities in RAC, with weight gain being a greater concern than weight loss. These findings emphasise the lack of care staff's understanding of malnutrition, as the RACs were in a study a year prior, and it was revealed that almost one third of all residents had malnutrition.^25^ This study builds upon prior research where a cross‐sectional survey of aged care staff revealed that care staff require greater knowledge of the nutritional needs of residents, particularly in comparison with other aged care staff.^15^ Additionally, care staff being concerned for residents who are overweight is not surprising given a focus on overweight/obesity in Australian public health campaigns and the awareness of workplace health and safety risks in physically assisting residents who are overweight in RAC.^26^ As such, this study supports prior calls for dedicated nutrition training for care staff,^11^ particularly regarding nutrition requirements for residents with malnutrition. Nutrition education and training need to be embedded both within the curricula of aged care certificate qualifications and on‐the‐job training, which is tailored to the specific nutrition care needs of residents. Beyond the training of care staff, this study reinforces calls for increased attention and resources towards routine malnutrition in RAC.^11^, ^25^ Regular malnutrition screening can supports care staff inrecognising residents with malnutrition and understanding its prevalence in RAC.^12^, ^25^ Malnutrition screening in RAC, and specific nutrition training for care staff are imperative to ensure appropriate nutrition care is provided to residents with malnutrition and therefore contribute to reducing malnutrition prevalence in the aged care sector.

The findings of this study revealed that care staff have deep and genuine concern about the well‐being of residents, including those with malnutrition. A recent study highlighted the importance of care staff to build strong, and supportive relationships with residents by prompting personalised care.^27^ Supportive relationships between care staff and residents have been associated with positive care outcomes and improved quality of life.^28^ The link between relationships and care was a sentiment voiced by the participants in the present study, who connected supportive care of residents to increased nutritional intake. While care staff recognised the importance of delivering meaningful care to residents, care staff reported facing barriers to providing this care. One key barrier identified was a lack of time allocated to providing care to residents, particularly those with malnutrition who have higher care needs. Similarly, care staff in other studies have expressed a desire to create therapeutic relationships aligned with person‐centred care principles but struggle to do so due to time constraints.^28^, ^29^ These findings reinforce the challenges faced by care staff in balancing their desire to form meaningful connections with residents and provide genuine care while meeting the demands of their professional responsibilities. Future research should explore strategies to enhance care staff capacity to deliver care they perceive as important, while staying within the bounds of their role.

To improve care outcomes of residents with malnutrition and reduce the high prevalence of malnutrition, changes must be made at the policy level. Beyond time constraints within their role, care staff acknowledged the organisational and policy impacts of inadequate staffing in their RAC, which hindered their ability to provide adequate care to residents, particularly those with malnutrition. Inadequate staffing in aged care is not a new finding, as it has been previously reported to be widespread across the aged care sector.^30^, ^31^ With inadequate staffing, the care staff in this study expressed similar feelings to that reported in earlier research, where care staff felt overworked due to understaffing and recognised the impact understaffing has on residents' care.^28^ While there have been recent initiatives delivered that focus on providing adequate support to care staff to care for residents,^32^ a specific focus on malnutrition is needed. A targeted strategy could include incorporating a validated malnutrition screening tool in the Australian National Aged Care Classification (AN‐ACC) Assessment Tool, similarly to how the AN‐ACC incorporates frailty.^33^ With the inclusion of malnutrition in this tool, RAC sites will be provided with more care funding to support residents with malnutrition.^33^ Targeted funding will provide care staff with more dedicated time to provide care to residents with malnutrition, a core finding recognised by care staff in this study.

Care staff in this study identified that residents lack social support. Lack of social support was linked by care staff to lower motivation to eat by residents, thereby contributing to malnutrition. In fact, this perception of care staff regarding the link between social support and malnutrition is well‐supported by research conducted with older adults across different settings.^28^ Research reveals that lower social engagement and less social support are associated with a higher risk of malnutrition.^28^, ^34^, ^35^ The recognition of the need by care staff for greater social connection for residents and increased family involvement has also been identified within the literature.^28^ Moreover, family participation in mealtime activities has been shown to improve residents' food intake.^28^ To improve residents' social support and reduce the reliance of care staff to provide social support, strategies to engage family members in RAC are warranted.^28^ Greater social support provided to residents, especially by family members, may contribute to improved nutrition intake and reduce the malnutrition risk of residents living in RAC.

The present study has many strengths, such as the use of rigorous methods employed throughout each stage of the research process and the use of the SEM framework to provide new valuable insights into care staff perspectives regarding malnutrition in the RAC. Additionally, this study builds upon existing research that has utilised the SEM framework^17^ in research design for studies conducted in RAC settings. As such, this study further supports the use of the SEM framework to explore the multideterminant factors relating to providing care to residents in the RAC sector. While there are many strengths to this study, some limitations exist. The transferability of the research findings needs to be carefully considered, given the small sample size of care staff, who were all from three privately owned RAC sites located on the east coast of Australia. This study was conducted in RAC sites that were previously involved in a research project by one researcher of the team (M‐C.O), posing a potential bias. To explore this potential bias, care staff were asked at the end of the focus group about their awareness of the prior project. Care staff acknowledged that they knew a research project had been conducted, but were unsure of the project's purpose, its findings or the student dietitian's role in visiting the RAC site. Therefore, the potential risk of bias appears low.^36^ Funding constraints resulted in only three focus groups being conducted, with 15 care staff involved. While this is a potential limitation, it is unlikely that additional focus groups would have yielded different results given the homogeneous nature of the findings. Time and location constraints beyond the research team's control may have impacted the findings, as two of the three focus groups were conducted via Microsoft Teams™, no participant checking of transcripts occurred, and the focus groups were relatively short in duration (34 min). The researchers also acknowledge a recent paper, which highlighted the challenges of conducting research in aged care.^36^ To gain a more comprehensive understanding of the topic, further research on a larger scale conducted in diverse RAC settings (i.e. public, private and not‐for‐profit) across Australia is required.

## 5 CONCLUSIONS

This study provided the first insight into care staff's understanding of malnutrition and exploring their perspectives on the multideterminant factors associated with providing nutrition care to residents. There are many opportunities to build the capacity of the care staff workforce to ensure adequate care is provided to residents with malnutrition. Implementing malnutrition screening in RAC and incorporating nutrition content in both vocational qualifications and on‐the‐job training, is imperative to enhancing care staff's understanding of malnutrition and their awareness of malnutrition prevalence in RAC settings. To support care staff in providing the genuine care they perceive residents deserve, initiatives to engage family in providing social support to residents, and sufficient care staff time and adequate staff are needed. Changes at the policy level, such as incorporating malnutrition in the AN‐ACC Assessment Tool and mandating malnutrition screening, are imperative to support care staff's understanding of malnutrition and to allow sufficient time to provide care to residents. Strengthening care staff capacity to meet residents' care needs is crucial for reducing the high prevalence of malnutrition in RACs.

## FUNDING INFORMATION

Financial support from Griffith University was received. The funders had no involvement in the study.

## CONFLICT OF INTEREST STATEMENT

No conflicts of interest declared.

## Data Availability

The data that support the findings of this study are available on request from the corresponding author. The data are not publicly available due to privacy or ethical restrictions.
